# Predicting life satisfaction based on the emotion words in self-statement texts

**DOI:** 10.3389/fpsyt.2023.1121915

**Published:** 2023-03-09

**Authors:** Mengyao Song, Nan Zhao

**Affiliations:** ^1^CAS Key Laboratory of Behavioral Science, Institute of Psychology, Chinese Academy of Sciences, Beijing, China; ^2^Department of Psychology, University of Chinese Academy of Sciences, Beijing, China

**Keywords:** life satisfaction, self-statement, machine learning, emotion, lexicon

## Abstract

Measuring people's life satisfaction in real time on a large scale is quite valuable for monitoring and promoting public mental health; however, the traditional questionnaire method cannot fully meet this need. This study utilized the emotion words in self-statement texts to train machine learning predictive models to identify an individual's life satisfaction. The SVR model was found to have the best performance, with the correlation between predicted scores and self-reported questionnaire scores achieving 0.42 and the split-half reliability achieving 0.939. This result demonstrates the possibility of identifying life satisfaction through emotional expressions and provides a method to measure the public's life satisfaction online. The word categories selected through the modeling process were happy (PA), sorrow (NB), boredom (NE), reproach (NN), glad (MH), aversion (ME), and N (negation + positive), which reveal the specific emotions in self-expression relevant to life satisfaction.

## 1. Introduction

Life satisfaction refers to an individual's subjective assessment of their quality of life based on criteria set by themselves. It is a comprehensive judgment of their family, work, and social life (1). As a core cognitive component of subjective wellbeing, life satisfaction is often seen as a valid measure of subjective wellbeing (2) and is proven to have a great impact on many aspects of an individual's mental health (3–5). Researchers found that life satisfaction is negatively correlated with depression and that improving life satisfaction can reduce depressive symptoms (6, 7). Yao et al. (8) found that life satisfaction is also negatively associated with anxiety. Suldo and Huebner (9) empirical evidence suggested that life satisfaction buffers against the negative effects of stress and the development of psychological disorders. Koivumaa-Honkanen found that low life satisfaction predisposes an individual to take their own life and has a long-term effect on suicide risk (10). Fergusson et al. (11) found that life satisfaction influences an individual's mental health. In addition, life satisfaction is also an important parameter to measure the quality of life of individuals in a certain society (4). Wong et al. (12) data-based study showed that social policies had different levels of impact on the life satisfaction of individuals. Therefore, the survey of people's life satisfaction is of great value in the study and practice of mental health in many scenarios and in the policy-making to promote the public's wellbeing.

As an important cognitive index, methods of measuring life satisfaction have been developed. The most commonly used method is by questionnaire, such as Diener et al. (13) Satisfaction with Life Scale (SWLS). Although the scales have shown high validity in many studies (14–16), it would be expensive and inefficient to carry out a large-scale survey. Therefore, in large-scale studies, life satisfaction is often measured by directly asking about individuals' feelings regarding their life by *one* single question (17), which cannot reflect the whole measurement structure of life satisfaction. In addition, these self-report methods are usually not time-sensitive enough and can only be used for prospective studies rather than retrospective studies. For example, in the case of the COVID-19 pandemic, researchers could not predict when the outbreak would occur and, therefore, could not conduct comparative studies on people's life satisfaction before and after the outbreak.

Self-statement text is a form of linguistic expression in which individuals express their opinions, experiences, thoughts, and feelings from a first-person perspective, such as comments, diaries, autobiographies, and original microblogs. Whether online or offline, individuals' self-statement texts can reflect their identity, social relationships, emotional attitudes, and other important information (18). In recent years, with the help of natural language processing and machine learning techniques, researchers have started to build predictive models to recognize individuals' subjective wellbeing and satisfaction based on their self-statement texts. Li et al. (19) established a predictive model of subjective wellbeing based on the characteristics of Weibo users' network behavior, user information, and micro-blog text. Schwartz used LIWC and LDA to build a message-level model, a user-level model, and a conjunctive model to predict individuals' happiness and draw a happiness map based on the content people published on Twitter and Facebook (20). Chatterjee et al. (21) used text mining and machine learning methods for sentiment analysis of users' online comments to discover the factors affecting customer satisfaction and build a predictive model. Wang et al. (22) established a dictionary of park landscape characteristics and built a prediction model of park satisfaction through content analysis of Beijing Park reviews on Dianping. The aforementioned studies indicated the possibility of building a satisfaction recognition model through self-expression, and the features used for prediction were usually complex and relied on various techniques of natural language processing.

Many studies have shown that individuals' emotional experiences and expressions are closely related to their life satisfaction. Researchers used questionnaires to study the relationship between individuals' positive emotions, negative emotions, and life satisfaction and found that emotions can affect their life satisfaction (23, 24). Vine used LIWC to extract emotion vocabulary from people's stream-of-consciousness writing and blogs and found that there is an association between emotion vocabulary and wellbeing (25). Tov et al. (26) used LIWC in the content analysis of individuals' diaries to investigate the relationship between their emotions and life satisfaction. Kramer (27) analyzed the relationship between the differential use of positive and negative emotion vocabulary and life satisfaction among Facebook users and demonstrated that life satisfaction could predict whether a person's update status was positive or negative. These studies have proven that there could be a close relationship between individuals' emotional expression and life satisfaction. However, most of these studies only distinguished the valence (positive/negative) of emotion, with the relationship between the various types of emotion in expression and life satisfaction still unknown. In terms of the need for timely, large-scale measurement of life satisfaction, whether and how to predict life satisfaction using affective words in self-expression could also be a valuable question to explore.

Therefore, this study aimed to make use of the emotional expressions in personal self-statement texts to predict life satisfaction by building machine learning prediction models. Meanwhile, we also explored the relationship between life satisfaction and various specific emotional expressions in self-statement.

## 2. Methods

### 2.1. Participants and data collection procedure

A total of 264 graduate and doctoral students were recruited to voluntarily participate in the study (195 women and 69 men). After informed consent, the subjects were first asked to freely write a few paragraphs in Chinese (at least 300 words) about their recent situation or feelings, similar to the way they naturally express themselves on social media such as Weibo. Moreover, they were required to fill out a questionnaire with a design based on the SWLS scale and including gender information.

All phases of the study were subject to ethical approval by the Institutional Review Board of the Institute of Psychology, Chinese Academy of Sciences with the ethical approval number H16003.

### 2.2. Measurement tools

#### 2.2.1. Life satisfaction

This study used the Satisfaction with Life Scale (SWLS) Chinese version (13) to measure the level of life satisfaction of the participants. The SWLS questionnaire is a widely used instrument for measuring satisfaction with life with good reliability (Cronbach's α= 0.78) and validity (28). This questionnaire consists of five questions and is scored on a 7-point Likert scale, which could be summed up to a total score as the index of general life satisfaction. The higher the total score, the higher the subjects' life satisfaction.

#### 2.2.2. Emotional dictionary

This study used three sentiment word dictionaries to extract emotional features from subjects' self-statement text. (1) The Dalian Institute of Technology Sentiment Dictionary (29): this dictionary contains a total of 27,466 words. It divides emotional tendencies into seven major categories and 21 sub-categories. (2) The Micro-blog basic mood lexicon (30): this dictionary contains a total of 818 words, covering five basic social emotion categories—happiness, sadness, anger, fear, and disgust. (3) The Compound emotion word categories (31): this dictionary is the compound vocabulary of “negative word + emotion word.” It contains three categories in total: P (negation + negative), N (negation + positive), and Ne (neutral).

Finally, we obtained 29 word categories in total as the dimensions of emotion features, as shown in Table 1.

**Table 1:** Sentiment dictionary dimension.

**Serial number**	**Dimension**	**Example**
**Positive**		
1	Happy (PA)^a^	Joyful, cheerful, and smiling
2	Freefromcare (PE)^a^	Down-to-earth, forgiving, and with a clear conscience
3	Respect (PD)^a^	Respect, affection, awe
4	Commendation (PH)^a^	Handsome, excellent, and accommodating
5	Trust (PG)^a^	Trust, confidence, reliability
6	Favorite (PB)^a^	Baby, adored, loved
7	Wishes (PK)^a^	Longing, Blessing, Long Life
8	Glad (MH)^b^	Loving, intelligent, and well-meaning
9	P (negation + negative)^c^	No regrets, no violations, no worries
**Negative**		
10	Anger (NA)^a^	Angry, irritated, furious
11	Sorrow (NB)^a^	Sorrowful, bitter, grief-stricken
12	Disappointment (NJ)^a^	Despair, regret, discouragement
13	Guilt (NH)^a^	Guilt, repentance, and a sense of guilt
14	Panic (NI)^a^	Panicked, flustered, overwhelmed
15	Fear (NC)^a^	Timid, scared, frightened
16	Boredom (NE)^a^	Irritable, stifled, self-seeking
17	Abomination (ND)^a^	Disgusting, shameful, distracting
18	Reproach (NN)^a^	Dull, vain, and hard-hearted
19	Jealousy (NK)^a^	Jealous, jealousy, envy
20	Doubt (NL)^a^	Hyper-attentive, suspicious, paranoid
21	Surprise (PQ)^a^	Strange, miraculous, astonishing
22	Aversion (ME)^b^	Dirty, philandering, bulky
23	Anger (MA)^b^	Enemy, reactionary, invasion
24	Fear (MD)^b^	Assassination, death, viciousness
25	Sadness (MS)^b^	Down, sad, mournful
26	N (negation + positive)^c^	Not interesting, not very active, not good at
**Neutral**		
27	Miss (PF)^a^	Thoughts, longing, longing for each other
28	Shy (NG)^a^	Shyness, shame, discomfort
29	Ne (Neutral)^c^	Not active, not dead, not bored

^a^Source: Dalian Institute of Technology Sentiment Dictionary; ^b^Source: Micro-blog basic mood lexicon; ^c^Source: Compound emotion word category.

### 2.3. Feature extraction and selection

After word segmentation, the three sentiment word dictionaries were used on subjects' writing texts to calculate the word frequency of each emotion word category. Each subject's text was processed separately, and finally, 29 emotional features were extracted.

To examine the relationship between each emotion word category and life satisfaction, we first conducted analysis using the Pearson correlation coefficient. It was expected that there existed some redundant features that would weaken the prediction performance. Hence, features that did not contribute to making predictions were removed. In our study, we used the method of forward feature selection as the feature selection technique.

### 2.4. Model training and testing

With the selected features, we started to train the regression model for predicting life satisfaction. Due to the small sample size used in this study, the support vector regression (SVR) method that performs well on small datasets was conducted. At the same time, we also used linear regression (LR), multi-layer perceptron regressor (MLP), random forest (RF), and decision tree (DT) to train the prediction model to compare the performances of different algorithms and attempt to select a better model. All models were trained through 5-fold cross-validation.

To test the validity of the predictive models, we calculated the Pearson correlation coefficients, which were the correlation between the predicted life satisfaction results of the model and the scores of the questionnaire. Higher correlation coefficients indicate better effect of the predictive model.

To test the reliability of the models, we adopted the method of split-half reliability testing in psychometrics. Concretely, we made use of another self-statement dataset, the textual posts of 50 Sina Weibo active users, because the split-half reliability test required more text data for each subject. First, the Weibo posts within a year of each Weibo user were spliced together to generate a single piece of text. To ensure the homogeneity of the split texts, we conducted the odd–even split-half method. For each piece of the text, all the sentences were numbered in order, and then, we obtained the odd half text containing the odd-numbered sentences and the even half text correspondingly. However, we conducted our predictive models on the odd half text and the even half text separately. Finally, the model-predicted life satisfaction scores from the odd and the even half text were compared through Pearson correlation analysis, with the correlation coefficient as the indicator of split-half reliability.

In our study, feature selection and model training were performed through scikit-learn, and other statistical analyses were conducted through SPSS 26.0.

## 3. Results

### 3.1. Correlations between emotion words and life satisfaction

To find out the relationship between emotion word categories and life satisfaction, we conducted Pearson correlation analysis on the word frequencies and the life satisfaction score, and the results are shown in Table 2.

**Table 2:** Pearson correlation coefficients (r) between features and life satisfaction.

**Features**	**SWLS**
**Positive**	
Happy (PA)^a^	0.15^**^
Free from care (PE)^a^	0.06
Respect (PD)^a^	0.10^**^
Commendation (PH)^a^	0.09
Trust (PG)^a^	0.04
Favorite (PB)^a^	0.13^**^
Wishes (PK)^a^	0.02
Glad (MH)^b^	0.24^**^
P (negation + negative)^c^	−0.02
**Negative**	
Anger (NA)^a^	–0.12^**^
Sorrow (NB)^a^	–0.20^**^
Disappointment (NJ)^a^	−0.07
Guilt (NH)^a^	−0.06
Panic (NI)^a^	−0.02
Fear (NC)^a^	–0.11^**^
Boredom (NE)^a^	–0.21^**^
Abomination (ND)^a^	−0.03
Reproach (NN)^a^	–0.20^**^
Jealousy (NK)^a^	−0.05
Doubt (NL)^a^	−0.08
Surprise (PQ)^a^	−0.08
Aversion (ME)^b^	–0.21^**^
Anger (MA)^b^	−0.11^**^
Fear (MD)^b^	−0.05
Sadness (MS)^b^	–0.11^**^
N (negation + positive)^c^	–0.26^**^
**Neutral**	
Miss (PF)^a^	0.03
Shy (NG)^a^	0.03
Ne (Neutral)^c^	0.07

^**^Represents p < 0.01.

^a^Source: Dalian Institute of Technology Sentiment Dictionary; ^b^Source: Micro-blog basic mood lexicon; ^c^Source: Compound emotion word category.

As shown in Table 2, the positive emotion words were positively correlated with life satisfaction, and the negative emotion words were negatively correlated with life satisfaction. Among the positive words, the four categories of happy, respect, favorite, and glad were significantly correlated with life satisfaction with the highest correlation coefficient up to 0.24; among the negative emotions, the eight categories of anger, sorrow, fear, boredom, reproach, aversion, sadness, and N (negation + positive) were significantly correlated with life satisfaction with the highest correlation coefficient up to −0.26. Compared to the positive emotions, the negative emotions significantly correlated with life satisfaction with more word categories and relatively higher correlation coefficients.

### 3.2. Results of feature selection

After feature selection, the remaining features are shown in Table 3.

**Table 3:** Feature selection results.

**Method**	**Number of items retained**	**Retention features**
FFS	7	**PA**, NB, NE, NN, **MH**, ME, N(negation + positive)

Bold represents positive emotion.

As shown in Table 3, the seven categories of happy, sorrow, boredom, reproach, glad, aversion, and N (negation + positive) were selected. Compared with positive emotion words, more negative emotion word categories were present in this list. It seemed that, in the emotion words lexicon reflecting life satisfaction, the negative words were richer than the positive words.

### 3.3. Validity of models in predicting life satisfaction

Appropriate parameter settings are essential for training to generate a well-performing machine learning model (32). After the tuning process of model parameters, the kernel function of SVR was set to “rbf,” the regularization coefficient was set to 1, the kernel coefficient was set to 100, the dimension of the polynomial kernel function was set to 2, and the other parameters used the default values.

After the 5-fold cross-validation, the values of the Pearson correlation coefficients between the predicted scores of life satisfaction and the scores of the SWLS questionnaire are shown in Table 4.

**Table 4:** Pearson correlation coefficients between the predicted scores and the questionnaire scores of life satisfaction.

**Method**	***r***
SVR	0.42^**^
MLP	0.40^**^
LR	0.39^**^
RF	0.31^**^
DT	0.27^**^

^**^Represents p < 0.01.

As shown in Table 4, the correlation coefficients between the predicted scores and the SWLS scores most achieved medium level (>0.30), while the values were varied among different algorithms. The SVR model had relatively higher validity, with the correlation coefficient between the predicted value and the true value reaching 0.42. The correlation coefficients between the predicted scores of the MLP model and the questionnaire score could also reach 0.40. For the other two algorithms RF and DT, the indexes of model validity were relatively lower. The correlation coefficients between the predicted scores of the DT model and the questionnaire score were only 0.27.

### 3.4. Split-half reliability

As the SVR model performed best in predicting life satisfaction, we further tested the split-half reliability of this model. With 50 Sina Weibo users' posts, we divided each user's posts into two halves, according to the parity of the chronological order. The trained SVR model as described earlier was applied to the two halves of textual data, achieving two sets of predicted life satisfaction scores, and then, the Pearson correlation coefficient was calculated as the index of split-half reliability. The test result of the SVR model was quite high (*r* = 0.939, *p* < 0.001), showing that the prediction scores calculated by this model were highly correlated on different self-statement texts of the same individual.

## 4. Discussion

### 4.1. Feasibility of predicting life satisfaction by the emotion words in self-statement

The results of modeling (Table 4) in this study showed that it is possible to recognize individuals' life satisfaction based on the emotion words used in their self-statement texts. In this study, we built the prediction models with SVR, and the correlation coefficient between the predicted and real-life satisfaction scores was 0.42. This validity was considered to be acceptable and meaningful in the field of personality and social psychology (33). When tested on social media texts, the model had also shown high split-half reliability. Specifically, the present study used only the emotion word frequencies as the extracted features for prediction models rather than any other linguistic or behavioral features. Compared with the intricate combinations of diverse features in previous modeling studies, our findings demonstrated the effect of the emotion elements in recognizing life satisfaction and provided a more concise and comprehensible predictive model.

### 4.2. Performance of different models

The different performances of models in our results indicated which method was more suitable to build the life satisfaction prediction model based on the self-statement text. In terms of the validity of the model, the SVR-based models performed relatively better, and the correlation coefficient between predicted and real scores reached the top of our study. Compared with the other algorithms in this study, previous researchers have found that the SVR algorithm was relatively more stable and robust and performed well, especially in small datasets (34). It also reminded us that different machine learning algorithms have different characteristics; therefore, researchers should choose the appropriate machine learning algorithm according to their research tasks and the nature of the research data.

### 4.3. The valence of emotion and life satisfaction

The results of this study (Table 2) indicated that the positive emotion words were positively correlated with life satisfaction, and the negative emotion words were negatively correlated with life satisfaction. Previous research found that individuals' mood can influence their life satisfaction (23, 24), that is, the emotion words in the self-statement texts in our study reflected the individuals' true emotional experience, and the valence of these emotion words reflected the direction that the life satisfaction changed.

According to the results of correlation analysis (Table 2), compared with positive emotion words, negative emotion words had a higher correlation coefficient with life satisfaction. From the results of the feature selection (Table 3), there were more negative emotion words than positive emotion words among the selected features. It seemed to indicate that in self-statement texts, negative emotion words conveyed more information about life satisfaction and made a greater contribution to the prediction of life satisfaction. The result was consistent with Baumeister's finding, which showed that negative events, experiences, relationships, and psychological states affected our feelings more than positive factors (35). Furthermore, compared with positive emotions, negative emotions were more differentiated, and could be described with more words (26). This asymmetry was also reflected in the dictionary used in this study, where the negative emotions contained more categories compared with the positive emotions.

### 4.4. Relationships between specific emotion categories and life satisfaction

In this study, the seven emotional features of happy (PA), sorrow (NB), boredom (NE), reproach (NN), glad (MH), aversion (ME), and N (negation + positive) were finally selected for modeling (Table 3). As shown by the correlation results (Table 2), these seven features were also those with the highest correlation coefficients with life satisfaction. These results suggested that these emotion categories played an important role in reflecting life satisfaction, which may bring meaningful inspiration to our understanding of the relationship between specific emotions and life satisfaction.

#### 4.4.1. Happy and glad

The emotion word categories happy and glad have similar contents expressing happiness. In our study, individuals with higher life satisfaction would express more happiness. Happiness is an emotional experience when an individual feels good, which will affect the individuals' evaluation of all areas of social life. When individuals are happy, they are more likely to make positive comments. As the emotional component of subjective wellbeing, happiness is very closely related to life satisfaction (36). Zou et al. (37) research also showed that a person's level of life satisfaction had a strong positive correlation with his level of happiness.

#### 4.4.2. Sorrow

Individuals with lower levels of life satisfaction expressed more sorrow relevant words. Sorrow is a negative emotional experience caused by negative events such as loss and frustration. In this emotional state, individuals' perceptions, thoughts, and judgments tend to be more negative (38), and the evaluation of life satisfaction could be influenced. Moreover, studies had found that individuals in sad moods tend to attribute negative events to the external environment (39), thus reducing their evaluation of the external environment and producing a sense of dissatisfaction.

#### 4.4.3. Boredom

Individuals with lower levels of life satisfaction used more words expressing boredom, which is a common negative emotion. When in this state of mind, individuals may develop anxiety and depression, thus isolating them from positive emotions such as happiness and joy, which will inevitably reduce the level of individual wellbeing (40). Previous research indicated that boredom may reduce an individual's life satisfaction (41, 42). Work and hobbies can alleviate an individual's boredom, and more boredom may imply a relative lack of meaningful work and joyful hobbies, which leads to lower life satisfaction.

#### 4.4.4. Reproach and aversion

The categories reproach and aversion are both made up of the emotion words expressing refusal, rejection, and blame. Individuals in our study with lower life satisfaction mentioned these words more in self-statement texts. In the process of social interaction, comments from others play an important role in forming self-perception and self-experience (43). The usage of reproach or aversion words may mean that the individual experienced negative comments or rejection from others, which could reduce her/his self-esteem and then life satisfaction. Other research has found that the tendency to criticize and nitpick could reduce individuals' feelings of satisfaction (44, 45), which could also be a possible reason explaining the relationship between those words and life satisfaction.

#### 4.4.5. N (negation + positive)

Individuals with lower life satisfaction used more negative compound words (negation + positive), such as “not happy” and “not interesting.” The present study demonstrated that experiencing the opposite of general positive feelings did relate to individuals' life satisfaction. Further study is needed in the future to distinguish the specific categories of positive emotion here. In previous research, the use of negation had an emotion-regulating effect, which can effectively downregulate negative emotions and reduce the intensity of individuals' experience of emotions (46, 47). Therefore, it is also possible in our study that individuals with low life satisfaction may use this expression for self-emotional regulation.

As a folk perception, it is taken for granted that all kinds of positive and negative emotions in our self-statement could somewhat reflect our life satisfaction. However, our results indicated that not all emotion categories can reflect life satisfaction. In fact, the majority of both positive and negative emotion categories in our study showed no significant correlation with life satisfaction or contribution to the model prediction. These emotion categories include commendation, trust, wish, panic, disappointment, guilt, jealousy, doubt, and so on. The scope of emotion words in analyzing life satisfaction was greatly reduced and simplified by our study. Some previous studies found that commendation would increase life satisfaction (48), but we did not find significant relationships between commendation emotion words and life satisfaction. That might be because our data were the self-statement texts from Chinese participants, and in the relatively modest and subtle Chinese culture people do not often mention praise in self-expression. The underlying causes still need further exploration.

### 4.5. Value of emotion word-based life satisfaction prediction in application and research

Our study built a predictive model with good validity and reliability which could identify individuals' life satisfaction only through the emotion words in their self-statement. While self-statements are quite common on today's social media and easy to obtain, this prediction model could be a choice of a measurement method to detect social media users' life satisfaction. Compared with previous studies, our limited emotion word features as the input of the model are much easier to extract and understand. It would be particularly suitable for a fast, large-scale, cost-effective investigation of life satisfaction in online scenarios, serving the public mental health and social wellbeing promotion. In addition, through the prediction study, we revealed the relationship between specific emotion word categories and life satisfaction and obtained new knowledge of the linguistic characteristics reflecting life satisfaction. The emotion categories provide specific clues for further exploring the factors and mechanisms in real life determining individuals' life satisfaction. The general importance of emotion expressions in our study also suggested that it may be possible to develop life satisfaction detection methods through other modalities of emotion expression, such as facial expressions.

## 5. Limitations and future studies

Although this study proves that it is feasible to build a prediction model of life satisfaction based on self-statement text, there are still some deficiencies. First, the sample data size of this study was limited and the focused subject group was the young; data can be collected through multiple channels in subsequent studies to expand the scope of the subject group. Second, the study used only 29 emotion-related features and did not consider the possible effects of general demographic data. In future studies, more factors can be considered to further optimize the prediction model. Third, due to the data-driven method, we can only obtain the correlation between emotion words and life satisfaction rather than the causal relationship, which still requires the guidance or verification of theoretical knowledge and experimental research.

## 6. Conclusion

The purpose of this study was to explore the relationship between emotion expressions in self-statement and life satisfaction and test whether an individuals' life satisfaction could be identified based on the emotion words in self-statements. We found that the criterion validity and split-half reliability in predicting life satisfaction of the SVR model achieved a fairly good level, demonstrating that a prediction model could be established by analyzing self-statement text, and it is possible to measure social media users' life satisfaction through their public disclosure. The emotion word features selected through our study shed light on the relationships between emotion expressions and life satisfaction and implied the mechanism of the generation of life satisfaction.

## Data availability statement

The raw data cannot be made public due to ethical privacy restrictions. If necessary, we can provide feature data. Requests to access the datasets should be directed to NZ, zhaonan@psych.ac.cn.

## Ethics statement

The studies involving human participants were reviewed and approved by the Institutional Review Board of the Institute of Psychology, Chinese Academy of Sciences (H16003). The patients/participants provided their written informed consent to participate in this study.

## Author contributions

NZ proposed the conception of this study and collected the essential data for this study. NZ and MS jointly designed the experimental flow of this study, drafted, reviewed, and edited the manuscript. MS implemented the algorithm, performed the experiments, and result analysis. All authors contributed to the manuscript and approved the submitted version.
